# Mutations to Less-Preferred Synonymous Codons in a Highly Expressed Gene of *Escherichia coli*: Fitness and Epistatic Interactions

**DOI:** 10.1371/journal.pone.0146375

**Published:** 2016-01-04

**Authors:** David J. Hauber, Dennis W. Grogan, Ronald W. DeBry

**Affiliations:** Department of Biological Sciences, University of Cincinnati, Cincinnati, Ohio, United States of America; Tel Aviv University, ISRAEL

## Abstract

Codon-tRNA coevolution to maximize protein production has been, until recently, the dominant hypothesis to explain codon-usage bias in highly expressed bacterial genes. Two predictions of this hypothesis are 1) selection is weak; and 2) similar silent replacements at different codons should have similar fitness consequence. We used an allele-replacement strategy to change five specific 3rd-codon-position (silent) sites in the highly expressed *Escherichia coli* ribosomal protein gene *rplQ* from the wild type to a less-preferred alternative. We introduced the five mutations within a 10-codon region. Four of the silent sites were chosen to test the second prediction, with a CTG to CTA mutation being introduced at two closely linked leucine codons and an AAA to AAG mutation being introduced at two closely linked lysine codons. We also introduced a fifth silent mutation, a GTG to GTA mutation at a valine codon in the same genic region. We measured the fitness effect of the individual mutations by competing each single-mutant strain against the parental wild-type strain, using a disrupted form of the *araA* gene as a selectively neutral phenotypic marker to distinguish between strains in direct competition experiments. Three of the silent mutations had a fitness effect of |s| > 0.02, which is contradictory to the prediction that selection will be weak. The two leucine mutations had significantly different fitness effects, as did the two lysine mutations, contradictory to the prediction that similar mutations at different codons should have similar fitness effects. We also constructed a strain carrying all five silent mutations in combination. Its fitness effect was greater than that predicted from the individual fitness values, suggesting that negative synergistic epistasis acts on the combination allele.

## Introduction

Synonymous, or silent mutations, *i*.*e*., those nucleotide mutations within a codon in a protein-coding gene that do not change the codon’s amino acid translation, were originally thought to be selectively neutral [1,2]. The landmark work of Ikemura [3–6] showed both that synonymous codons are not used at random in many genes and that the cytoplasmic abundance of different iso-accepting tRNAs are correlated with codon-usage frequency. Additional work showed that codon-usage bias is more pronounced in highly expressed genes [7–9] and is present in a wide variety of organisms [8,10–13]. This evidence led to the hypothesis that both codon-usage bias and tRNA abundances are controlled by natural selection, with the selection presumed to be quite weak. A general view emerged that codon usage in highly expressed genes is determined by interactions among the effects of mutation, weak selection and drift [14–17]. Underpinning the mutation-selection-drift view is the inference that selection on any particular silent site in a highly expressed gene is a direct result of either the codon’s effect on transcription initiation, as mediated by the codon’s effect on mRNA secondary structure (for sites in the region +4 to -37 relative to the initiation codon; [18]), or the codon’s effect on translation efficiency, as mediated by the interaction between the codon and the tRNA(s) used by the cell to translate it (in the remainder of the mRNA).

Recent data challenge the idea that synonymous codon usage outside of the initiation region is controlled exclusively by mutation-weak selection-drift balance based on codon-tRNA coevolution. Lind et al. [19] engineered a collection of mutants in two ribosomal protein genes and expressed them in the bacterium *Salmonella typhimurium*. They found that the distribution of fitness effects of synonymous mutations was similar to that of non-synonymous mutations, with measured selection coefficients as large as 0.0279 operating against several individual silent mutations. The efficacy of selection (*vis-à-vis* drift) is a function of the selection coefficient and the population size (N), with selection being almost completely effective at fixation of the favored allele when 4Ns > 10 [20]. For s > 0.02, selection is strong for any N > 125, which is likely to hold for both *S*. *typhimurium* and *E*. *coli*. In a very different kind of study in a multicellular organism, Lawrie et al. [21] examined genome sequences generated for 130 homozygous strains derived from a single population of *Drosophila melanogaster*. They inferred the strength of selection on 4-fold degenerate silent sites by comparing the allele-frequency spectra of those sites to the spectra of matched nearby sites in short introns, which were assumed to represent the allele-frequency spectrum expected under strict neutrality. They found that approximately 21% of all 4-fold degenerate silent sites in exons show evidence of strong purifying selection, with selection coefficients ranging up to 0.02 –a value similar to those reported for *Salmonella* by Lind et al. [19]. It is difficult to reconcile observed selection coefficients of that magnitude with the hypothesis that selection acts primarily through the effects of codon-tRNA interaction, because strong selection would lead to universal usage of the preferred codon, rather than codon-usage bias.

In the work reported here, we assembled an experimental system in *Escherichia coli* strain K-12 with which we can insert specific silent mutations. The rapid growth typical of *E*. *coli* allows for many generations in a short period of time, allowing us to discriminate between strains differing in fitness by a selection coefficient as small as ~1x10^-3^. We introduced silent mutations into the highly expressed gene *rplQ*, which encodes the 50S ribosomal subunit protein L17. *rplQ* is transcribed as the downstream-most unit of the alpha operon [22] and is translationally regulated by binding of S4 protein to a conserved mRNA secondary structure element that extends to approximately 85 nt from the initiation codon [23]. We chose codons to mutate in order to test a prediction that arises from the hypothesis that codon usage bias is a result of selectively driven coevolution between codon usage and the pool of available tRNAs, while avoiding the regulatory element at the 5’ end of the mRNA. We hypothesize that selection against a particular uncommon codon should be independent of the codon’s position in the coding sequence (so long as the codons are well away from the site of translation initiation). If we introduce “matching” mutations—mutations from the same wild-type silent site to the same mutant silent in two different codons that encode the same amino acid—then we expect both mutations to have quantitatively similar fitness effects. We introduced two pairs of matching mutations (a closely linked pair of leucine codons and a closely linked pair of lysine codons) and, in both cases, the prediction of similar selective effects is not upheld. We also provide evidence for negative synergistic epistasis for fitness when five mutant silent sites are combined into one allele.

## Materials and Methods

### Preparation of an Ara^-^ marked strain

We created a defined *araA* mutant of the MG1655 strain of *E*. *coli* K-12, using an allele-replacement strategy modified from Hamilton et al. [24] and Blomfield et al. [25], as follows. A portion of the *araA* gene (positions 67320 through 68214 of the MG1655 genome; GenBank U00096; Blattner et al. 1997) was amplified with primers *araA*O+ and *araA*O- (Table A in S1 File) and inserted into the plasmid pCR^®^-Blunt II-TOPO^®^. Digestion of that plasmid with endonuclease *Bcg* I releases a 32-bp fragment with unique 2-bp 3’ overhangs from the cloned *araA* segment, which was then replaced with a corresponding synthetic, double-stranded oligonucleotide designed to introduce a stop codon, a 2-bp deletion, and a *Bam*H I recognition site into *araA* (Table A in S1 File). *Pst* I digestion was then used to transfer this modified *araA* sequence to the conditional-replication (temperature-sensitive, Ts) plasmid pIB307 [25]. Table B in S1 File summarizes all plasmid derivations in this paper.

Chemically competent MG1655 cells were transformed with the Ts plasmid carrying the mutated *araA* segment at 30°, and chloramphenicol-resistant (Cm^R^) colonies were isolated. A culture of approximately 10^8^ of these cells in 20 mL of Luria-Bertani Medium (LB) supplemented with chloramphenicol (60 μg/mL) was then incubated at 42°, the restrictive temperature for pIB307. Only those cells in which the plasmid had integrated into the host genome can propagate under these conditions. Clones in which integration occurred by homologous recombination at the *araA* locus were identified by their Ara^-^, Cm^R^ phenotype using replica plating on media described below; these clones have the wild-type *araA* gene interrupted by the plasmid bearing the mutated *araA* gene.

As a result of the duplication flanking the integrated plasmid, one of the *araA* alleles and the plasmid can be excised by a spontaneous second homologous recombination event. Some fraction of these excision events will remove the wild-type *araA* sequence, leaving the mutant *araA* sequence in the genome [25]. To isolate cells in which plasmid excision had occurred, we used a variant of penicillin enrichment [26,27] to eliminate chloramphenicol-resistant clones from the population, based on the principle that chloramphenicol stops the growth of chloramphenicol-sensitive cells, but does not kill them; ampicillin, in contrast, kills only growing cells, which, under conditions of the enrichment, are the chloramphenicol-resistant clones.

The cells of the strain with the integrated plasmid were allowed to grow in 20 mL of LB without chloramphenicol at 42°C to approximately 2–4 × 10^8^ cells/mL. Chloramphenicol (60 μg/mL) was then added to halt growth of cells lacking the plasmid, and one hour later (at approximately 6–8 × 10^8^ cells/mL) ampicillin (100 μ/mL) was added to kill actively growing cells [28,29]. Additional chloramphenicol (30 μg/mL) was added with the ampicillin to keep its level sufficiently high. After 1–2 hours, the remaining cells were pelleted by centrifugation, washed in water, and transferred to 20 mL fresh LB for a second round of this enrichment.

Chloramphenicol-sensitive (Cm^S^), *araA*^*-*^ cells were identified by replica plating on LB/chloramphenicol and LB/arabinose/TTC medium (LB with 1.5% agar, 5% L-arabinose, and 0.0015% triphenyltetrazolium chloride). On the latter plates, Ara^+^ colonies have the normal cream color, but Ara^-^ colonies appear pink [30,31]. One pink colony was chosen, and the *araA*^*-*^ allele was confirmed by sequencing a PCR product obtained with primers *araA*F3 and *araA*R3 (located within the *araA* gene but outside the cloned region (Table A in S1 File).

### Cloning and mutagenesis of the *rplQ* gene

**Fig 1:**
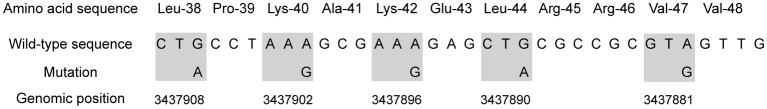
Silent mutations generated for this study. The five mutations introduced into the wild-type *rplQ* gene *en bloc* to create the *rplQ*m5 allele and singly to create the *rplQ*mL44, *rplQ*mL38, *rplQ*mK40, *rplQ*m42, and *rplQ*mV47 alleles are indicated. The region shown is the segment that is excised by digestion of *rplQ* with *Bcg* I. Codons in which the silent mutations were placed are shaded. Positions in the *E*. *coli* MG1655 genomic sequence (Genbank accession U00096) are shown for each mutant nucleotide.

A fragment spanning positions 3437451–3438272 of the MG1655 sequence (GenBank accession U00096) was amplified with primers *rplQ*+ and *rplQ*- (Table A in S1 File) and cloned into the plasmid pCR^®^-Blunt II-TOPO^®^. The cloned fragment includes the complete *rplQ* coding sequence, along with flanking sequences of approximately 250 bp upstream and 185 bp downstream. Digestion with *Bcg* I released a 32bp fragment with 2bp overhangs that included the codons that specify amino acids 38–47 of the L17 ribosomal protein. We designed six different duplex oligonucleotides (Integrated DNA Technologies, Coralville, Iowa) with the appropriate complementary overhangs. Five had sequence identical to the wild type except for a single, third-position silent mutation that produced one of the five mutations used in this study: in leucine-encoding codons L38 and L44 the wild-type CTG was replaced with CTA; in lysine-encoding codons K40 and K42 the wild-type AAA was replaced with AAG; and in the valine-encoding codon V47 the wild-type GTA was replaced with GTG (Fig 1). Relative synonymous codon usage (RCSU) values for highly expressed genes in *E*. *coli* for these codons are: 5.33 (CTG wild-type) *vs*. 0.04 (CTA mutant) for the two leucine codons, 1.60 (AAA wild-type) *vs*. 0.40 (AAG mutant) for the two lysine codons, and 1.11 (GTA wild-type) *vs*. 0.50 (GTG mutant) for the valine codons [11]. The sixth oligonucleotide was used to create a strain with all five mutations present together, and was identical to the wild-type sequence except for third-position silent mutations that create the same five mutations introduced into the single-mutant plasmids. We separately ligated each of the six oligomers into the site vacated by the *Bcg* I fragment in the plasmid carrying the wild-type *rplQ* sequence. This resulted in a series of high-copy plasmids, each carrying a mutated *rplQ* allele. Sequencing of PCR products with M13 primers verified the presence of the correct mutation in each of these plasmids. The resulting mutated *rplQ* alleles were transferred, along with the associated flanking sequences, from the high-copy plasmids to the Ts pIB307 as a 934-bp *Hin*d III—*Xba* I fragment, generating a series of Ts-*rplQ*m (mutated *rplQ*) plasmids (Table B in S1 File).

### Allele replacement at the *rplQ* locus

Chemically competent, Ara^-^ cells were separately transformed with one of the Ts-*rplQ*m plasmids, and Cm^R^ colonies were isolated. Clones in which the Ts-*rplQ*m plasmid had integrated into the bacterial chromosome were isolated as described above. Unlike *araA*, transcription of the *rplQ* gene was not interrupted by the plasmid because the whole gene was cloned along with flanking sequences. Integration within the cloned *rplQ* segment was verified by PCR using primer pairs pIB307F1 / *rplQ*F3 and pIB307R1 / *rplQ*R3. Primers pIB307F1 and pIB307R1 anneal within the pIB307 vector sequence, while *rplQ*F3 and *rplQ*R3 anneal in the *E*. *coli* genome, but outside the *rplQ* segment that was originally cloned (Table A in S1 File). One Cm^R^ clone for each of the single-mutant alleles and three separate clones for the quintuple-mutant allele (referred to below as *rplQ*m5e, -h, and—i; note that these clones are derived from a single insertion event but separate excisions) were retained for analysis. Excision of the Ts plasmid was selected as described above, and presence of the correct mutant allele in each clone was identified by PCR and DNA sequencing.

### Reverting the quintuple-mutant allele to wild-type

The *Xba* I / *Hin*d III fragment from the pCR^®^-Blunt II-TOPO^®^-based plasmid carrying the wild-type *rplQ* sequence was cloned into pIB307, forming a Ts-*rplQ*wt plasmid. Chemically competent cells of the strain carrying the *rplQ*m5i allele (one of the three strains carrying the quintuple-mutant allele) were transformed with the Ts-*rplQ*wt plasmid. Chromosomal integration of the Ts-*rplQ*wt plasmid was selected as described above, and a Cm^S^ colony derived from one integrant was confirmed to have the wild-type *rplQ* sequence by sequencing.

### Competition Experiments

Competitions always included one Ara^+^ and one Ara^-^ strain. Five to 12 replicate cultures (20 mL LB medium in 50-mL Erlenmeyer flasks) per experiment were inoculated with 100 μL of corresponding independent cultures grown overnight in LB medium. Initial population sizes (N_0_) for both strains in each competition were determined by serial dilution and plating on LB/arabinose/TTC plates as described above, using colony color to distinguish between Ara^+^ and Ara^-^ colonies (the flasks were chilled to prevent growth during this procedure). N_0_ was typically between 2.5 x 10^8^ and 5.0 x 10^8^ cells. Flasks were incubated at 37°C with orbital shaking at ca. 200 rpm for a growth period of 3–4 hours. At the end of this period, the population sizes of the two strains were determined for one replicate by plating on an LB/arabinose/TTC plate, and another growth period was initiated for all replicates by transfer of 100-μL from the previous flask into 20 mL of fresh LB (1:200 dilution). This cycle continued until there was approximately a 2-fold difference in the cell density of the two strains, or until a total of 14 growth periods (representing over 100 effective doublings of the populations) had been completed. Replicate cultures were rotated with respect to transfer sequence and position in the incubator. Growth of *E*. *coli* is halted at 2° [32,33], so refrigeration at 2° was used to suspend growth when necessary (overnight, weekends).

Relative fitness of the two strains was determined from initial (N_0_) and final (N_f_) population sizes. Fitness for each replicate was calculated as *w* = *D*_*m*_*/D*_*wt*_, where D_m_ and D_wt_ represent the number of doublings of the mutant and wild-type strains, respectively. D_m_ and D_wt_ were calculated as *D = log*_*2*_*(N*_*f*_* / N*_*0*_*)*. N_f_ was calculated as the final colony counts per milliliter multiplied by the culture volume and total dilution factor, i.e., 20·(200)^tr^ where tr is the number of transfers for each mixed culture. Mathematically, this is identical to the ratio of their respective Malthusian parameters [30]. The average fitness across replicates was used as the strain’s fitness.

Negative, synergistic epistasis would be considered to be occurring if the measured relative fitness for the quintuple-mutant strain is lower than the fitness projected from the measured relative fitnesses of the 5 separate single-mutant strains. The projected fitness *w* was calculated by both the multiplicative formula,$w={\prod_{i=1}^{n}\left(1+s_{i}\right)}^{n}$, and by the additive formula,$w=1+\sum_{i=1}^{n}s_{i}$, in which s_i_ is the individual selection coefficient values for each single mutation [34]. To determine statistical significance of any difference between predicted and observed fitness values for the quintuple-mutant strains, we used an approximation for the propagation of error for each calculation to estimate the variance of the resultant expected fitness. The variance approximation, V_w_, for the accumulated fitness effects was calculated as

$$V_{w}={\sum_{}^{}\left(\frac{\partial w}{\partial n}\right)}^{2}V_{n},$$ (1)

where V_n_ is the variance for each individual fitness value and $\frac{\partial w}{\partial n}$ is the partial differential with respect to each individual fitness variance (see Info A in S1 File). This approximation is considered valid for any function *w = f(x*, *y*, *z*, *…)* for which the individual variances are not great [35,36].

## Results

### Neutrality of the *araA*^*-*^ marker

**Fig 2:**
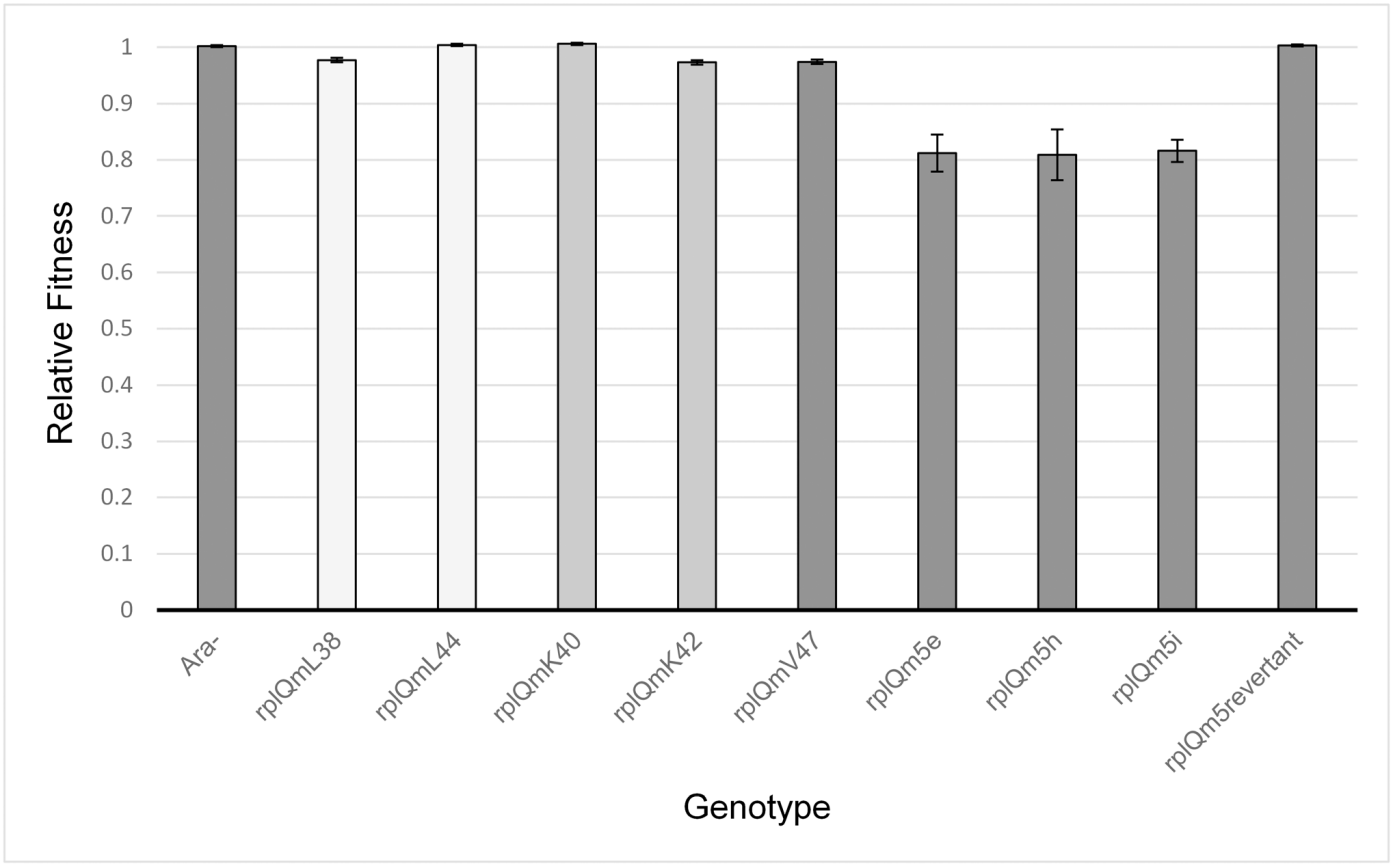
Fitness of mutant strains as determined from competition experiments against wild-type *E*. *coli* K-12 MG1655. Error bars denote one standard deviation for each experiment.

Our allele replacement strategy was designed so that we could introduce a single- or multiple-base mutation into the *E*. *coli* chromosome while making no other changes to either the *rplQ* gene or to any other region of the genome. Prior to measuring fitness, we cured the mutated strains of the exogenous plasmid used to deliver the mutant constructs. In order to measure fitness by direct competition against the wild type, we marked the mutant strains by inactivation of the *araA* gene. On appropriate indicator media, the ability or inability to catabolize arabinose (Ara^+^ or Ara^-^ phenotype, respectively) provides a selectively neutral marker for distinguishing two *E*. *coli* strains in competition experiments [30,31,37]. To confirm the suitability of the visually scorable Ara phenotype as a neutral marker under the conditions of our competition experiments, we performed preliminary experiments in which our engineered Ara^-^ strain was in competition with the wild-type strain MG1655. In the preliminary competition experiment with the largest number of replications, a fitness of 1.002 ± 0.002 was obtained (Table 1 and Fig 2). Combining all the preliminary competitions (Tables D-G in S1 File) gives a fitness of 1.0009 ± 0.0021 for the Ara^-^ strain. For the competition in Table G in S1 File, we calculated the minimum detectable difference for *α = 0*.*05* and *β = 0*.*1*[38] as 0.17 doublings over 105 doublings (Info B in S1 File). Detailed results from all competition experiments can be found in Tables D-P in S1 File.

**Table 1:** Total effective doublings (± 1 s.d.), relative fitness values (± 1 s.d.) and selection coefficients for the mutant genotypes constructed in this study, based on competition experiments between the Ara^-^, *rplQ* substrains and the wild-type *E*. *coli* K-12 substrain MG1655.

Genotype	Doublings	Relative Fitness	Selection Coefficient
Ara-	105.045 ± 0.106	1.002 ± 0.002	-0.002
wild type	104.841 ± 0.139	1	
*rplQ*mL44; Ara^-^	105.63 ± 0.16	1.004 ± 0.002	-0.004
wild type	105.19 ± 0.14	1	
*rplQ*mL38; Ara^-^	35.68 ± 0.15	0.977 ± 0.004	0.023
wild type	36.53 ± 0.23	1	
*rplQ*mK40; Ara^-^	105.18 ± 0.14	1.006 ± 0.002	-0.006
wild type	104.58 ± 0.14	1	
*rplQ*mK42; Ara^-^	49.89 ± 0.14	0.973 ± 0.004	0.027
wild type	51.26 ± 0.13	1	
*rplQ*mV47; Ara^-^	50.27 ± 0.11	0.974 ± 0.004	0.026
wild type	51.62 ± 0.16	1	
*rplQ*m5e; Ara^-^	4.56 ± 0.23	0.812 ± 0.033	0.188
wild type	5.61 ± 0.12	1	
*rplQ*m5h; Ara^-^	4.85 ± 0.19	0.809 ± 0.045	0.191
wild type	6.01 ± 0.18	1	
*rplQ*m5i; Ara^-^	4.68 ± 0.13	0.816 ± 0.020	0.184
wild type	5.74 ± 0.12	1	
reverted wild-type *rplQ*; Ara^-^	104.85 ± 0.20	1.003 ± 0.002	-0.003
wild type	104.24 ± 0.26	1	

Selection coefficients > 0 indicate selection against the allele. Data for individual replicates are in the supporting material.

### Fitness of the single-mutant *rplQ* strains

We engineered five separate single-base-mutation strains. Five silent sites were chosen, each located more than 100 nucleotides away from the initiation codon and all clustered within a 10-codon segment. We used these mutant strains to test a specific prediction of the codon-tRNA coevolution hypothesis—that the same silent mutation in different amino acid positions within the gene should have similar selective effects. To accomplish this we constructed two pairs of matching mutant alleles in which both members of each pair had the same wild-type silent site and to which we introduced the same less-preferred silent site. First, two different codons encoding leucine (L38 and L44) were changed from the wild-type CTG codon (*S*_*i*_ = 1.07; Xia 2015) to the less frequently used CTA (*S*_*i*_ = 0.13). Likewise, two different codons encoding lysine (K40 and K42) were changed from the wild-type AAA (*S*_*i*_ = 1.05) to the less frequently used AAG (*S*_*i*_ = 0.85). For the fifth single change, we altered one codon encoding valine (V47) from the wild-type GTA (*S*_*i*_ = 1.51) to GTG (*S*_*i*_ = 0.80). *S*_*i*_ is a measure of codon adaptation that accounts for mutation bias; values of *S*_*i*_ > 1 indicate codons with a selective advantage and values of *S*_*i*_ < 1 indicate codons at a selective disadvantage [39]. In addition to developing the *S*_*i*_ index, Xia [39] compiled values for all synonymous codons for *E*. *coli* strain K-12, which is used in this study. Finally, we constructed a single strain that incorporated all five mutations together. We used that strain to test for epistatic interaction.

Three of the five single-mutant strains exhibited lowered fitness relative to the MG1655 wild-type, with selection coefficients > 0.02, while the other two had fitness values that were slightly greater than that of the wild-type (Table 1 and Fig 2). The two matching mutations introduced into leucine-encoding codons had different fitness effects, and the two matching mutations introduced into lysine-encoding codons had different fitness effects. For the leucine-encoding codons, the *rplQ*mL38 (CTA) allele exhibited a selection coefficient of approximately 0.023 (relative fitness 0.977 ± 0.004) while the *rplQ*mL44 (CTA) allele had a selection coefficient of approximately -0.004 (relative fitness 1.004 ± 0.002). For the lysine-encoding codons, the *rplQ*mK42 (AAG) allele exhibited a selection coefficient of approximately 0.027 (relative fitness of 0.973 ± 0.004) while the *rplQ*mK40 (AAG) allele exhibited a selection coefficient of approximately -0.006 (relative fitness 1.006 ± 0.002). Finally, the *rplQ*mV47 (GTG) allele exhibited a selection coefficient of approximately 0.026 (relative fitness 0.974 ± 0.004). For all 5 single—mutation strains, the difference between the number of doublings during the competitions for the mutant strains compared to the wild-type MG1655 was significant (2-sample t test, P<0.001).

### Fitness of the quintuple-mutant strains

Three Ara^-^ *rplQ*m5 strains were retained (*rplQ*m5e, -h, and -i), and each of the three was used separately in competitions against the wild-type strain MG1655, thereby providing genetic replicates for these determinations. The measured fitnesses for the three *rplQ*m5 strains were 0.812 ± 0.033, 0.809 ± 0.045, and 0.816 ± 0.020 (Table 1 and Fig 2). The average fitness across the three strains was 0.812 (s = 0.188).

### Fitness of the *rplQ*wt revertant strain

To confirm that the fitness deficit of the quintuple-mutant *rplQ*m5 allele was due to the five silent mutations introduced into the *rplQ* gene, and not to some unknown mutation(s) elsewhere in the *E*. *coli* genome, we used the *rplQ*m5i strain as the starting material and used our allele-replacement technique to restore the wild-type *rplQ* sequence. The resulting *rplQ*wt-revertant strain had a relative fitness of 1.0029 + 0.0015, indicating that the fitness deficit seen in the *rplQ*m5i strain (Table 1 and Fig 2) was solely a function of the *rplQ* allele. While the two-sample *t* test indicated a significant difference in the number of doublings (0.002<P<0.005) between *rplQ*m5-revertant and the wild-type MG1655 in the competition experiment, the difference between the relative fitnesses of *rplQ*m5-revertant vs. MG1655 (*w* = 1.003 ±0.002) and the ancestral Ara^-^ marked strain vs. MG1655 (*w* = 1.002 ±0.002), was not significant, as determined by a 2-sample t test (0.10<P<0.20).

### Secondary structure of the mutant alleles

The mutant alleles’ potential for altering the secondary structure of the *rplQ* mRNA were estimated, based on ΔG values as calculated using the mfold server [40], with differences between each mutant allele and the wild-type being expressed as ΔΔG (Table 2). We find no clear relationship between the strength of selection and the magnitude of the change in folding free energy for the mutant vs. the wild-type alleles (Table 2). We did not generate enough mutant strains to allow for a statistical test of correlation between ΔΔG and s, but the pattern observed was not consistent with what would be predicted under a scenario in which large changes in folding energy result in substantial selection against an allele. The mutant allele with the largest |ΔΔG| was mK40, which had one of the two smallest selection coefficients (and which was under slightly positive selection compared to the wild-type). Two versions of the folding algorithm are offered on the mfold server, and they return substantially different ΔΔG values for all of our mutant alleles. With the older version 2.3 algorithm, all 3 mutations with large selective effect (mL38, mK42 and mV47) returned ΔΔG values < -3.9, but the newer version 3.0 returned ΔΔG = 0 for mK42 and ΔΔG > 0 for mL38 (Table 2).

**Table 2:** Change in folding energy (ΔΔG) of the *rplQ* mRNA for the five single-mutant strains generated in this study.

Strain	s	ΔΔG Quickfold 2.3	ΔΔG Quickfold 3.0
wild-type	0.000	0.00	0.00
mL38	0.023	-4.68	0.80
mK40	-0.006	-8.30	-3.50
mK42	0.027	-5.28	0.00
mL44	-0.004	-3.93	0.50
mV47	0.026	-6.55	-1.80

Selection coefficients are from Table 1.

### Epistasis

**Fig 3:**
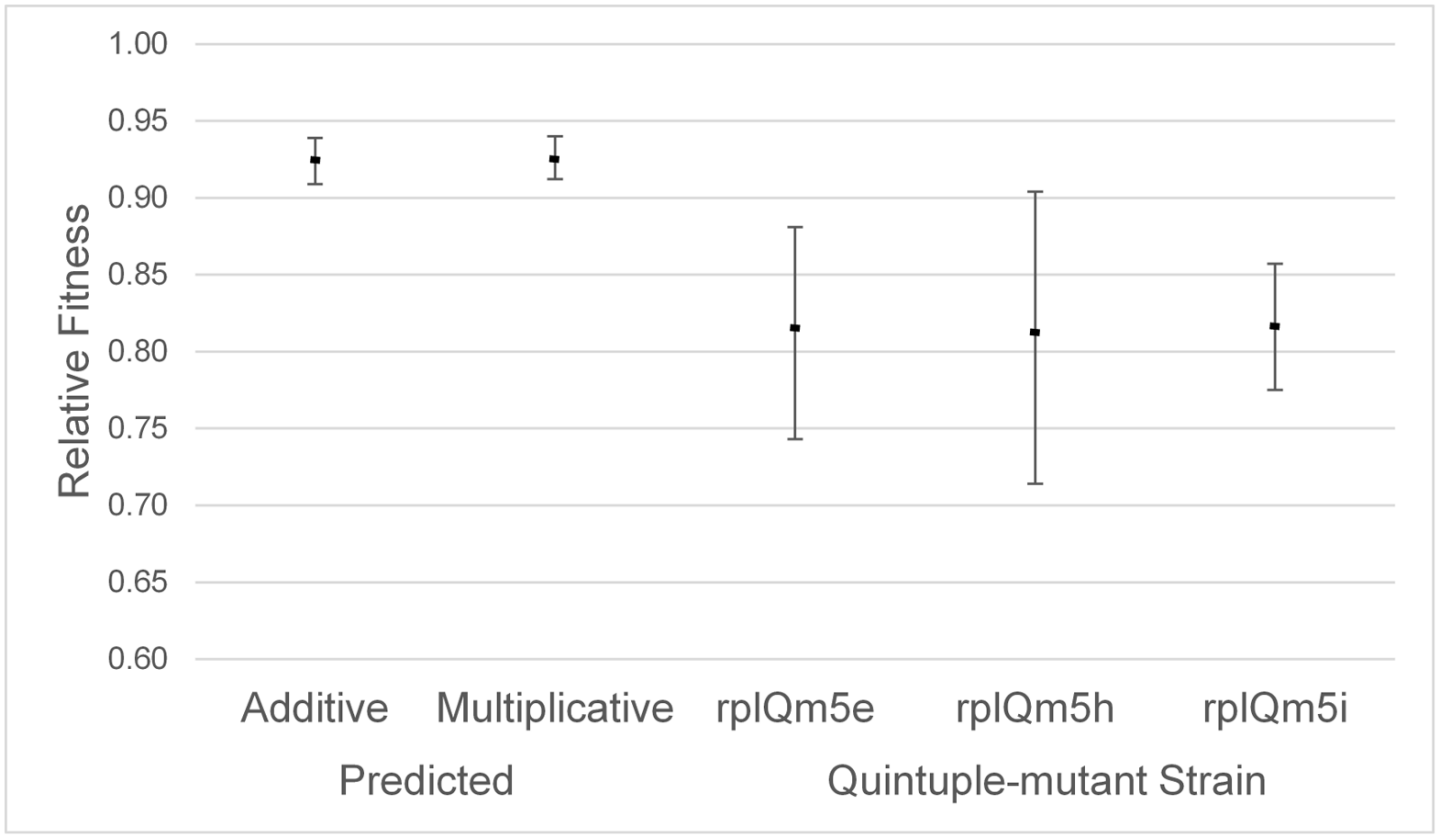
Predicted and observed fitness of the quintuple-mutant allele. Error bars indicated ± two standard deviation units (for the predicted fitness) or the 95% confidence interval (for the observed fitnesses). The predicted fitness for the quintuple-mutant strain was calculated after assigning a fitness of 1.0 to the two single-mutant strains with observed fitness > 1.0, but with the observed among-replicate variance for all five single-mutant alleles being used to calculate the standard deviation of the predicted fitness.

Based on the fitness values measured for each of the five single-mutant alleles, we predicted the combined fitness effect expected for an allele containing all five mutants together, using either multiplicative or additive fitness. These predicted combined fitness values are 0.935 (multiplicative) and 0.934 (additive). As a more conservative estimate, we assigned a fitness of 1 to the two individual mutations with measured fitness > 1.0. In that case, the expected values for the fitness for the quintuple-mutant strains were 0.926 (multiplicative) and 0.924 (additive). The observed fitness values for the quintuple-mutant strains ranged from 0.809 to 0.816, with an average fitness of 0.812, well below the lowest of the predicted fitness values (0.924) (Fig 3). In terms of selection coefficients, it would require five mutations each with s = 0.041 (multiplicative) or s = 0.037 (additive) to result in the observed value of s = 0.188 for the five mutations combined. The largest selection coefficient observed for any of the single mutations was s = 0.027 (*rplQ*mK42). The estimated standard deviation for our calculated combined-fitness values were ±0.008 (additive) and ±0.007 (multiplicative). These results are consistent with a conclusion that negative, synergistic epistasis is occurring among some or all of these five silent mutations within *rplQ*.

## Discussion

A long-standing hypothesis to explain the non-random usage of synonymous codons in highly expressed bacterial genes is that codon usage and tRNA pools co-evolve to maintain maximal levels of gene expression [17,41]. If codon-tRNA matching is the only factor controlling synonymous-codon usage, then one prediction that can be derived from that hypothesis is that identical selectively unfavored codons will have similar effects on fitness independent of their position within the coding sequence. Our data refute that prediction. We found that two closely linked lysine codons with matching silent mutations had very different fitness effects, as did two closely linked leucine codons with matching silent mutations. In addition to the large fitness differences between the matching mutations, our data also show fitness effects for three (of five) silent mutations that are much larger than what would be expected under a mutation-selection-drift model for the maintenance of codon-usage bias. Our conclusions are dependent on two assumptions. First, we assume that our allele-replacement procedure resulted in no changes beyond those intended. We partially tested that assumption by reverting one of the strains containing 5 mutations back to the wild-type sequence, and observing the expected rescue of fitness back to wild-type level. Second, we assume that the only function of the *rplQ* mRNA is to be transcribed into the L17 ribosomal protein. The data presented here, by Lind et al. [19], Lind & Andersson [42] and Lawrie et al. [21] all indicate that a subset of silent sites are under unexpectedly strong selection (with s > 0.02). Selection coefficients of that magnitude would produce strong purifying selection, unless the effective population size is very small. Nevertheless, the presence of a subset of silent positions that are under strong selection does not preclude the operation of codon-tRNA coadaptation selection on the remainder of the silent sites in those highly expressed genes, and it is possible that different forms of selection act on different silent sites. It appears, however, that strong selection on some fraction of silent sites may be the norm. Tests designed to discern the mechanisms behind the weaker codon-tRNA coadaptation effects should take into account the likelihood that some fraction of the silent sites will be essentially invariable at the scale of population-genetic tests for weak selection, due to strong stabilizing selection. For example, Xia [39] re-examined the results of Kudla et al. [43] and calculated that codon adaptation accounts for ~17% of the variation in protein production. It might be that codon adaptation would be an even stronger predictor of protein expression if the subset of strongly selected silent sites were first removed from consideration. Likewise, tests designed to use silent sites as a proxy for selectively neutral variation will be misled if a large fraction of silent sites are, in fact, subject to strong stabilizing selection Unfortunately, we do not yet understand the mechanism underlying strong selection on silent sites, nor do we know how to predict which sites will or will not be affected by strong selection.

### Comparison of our results to recent studies

Working with *E*. *coli*, Kudla et al. [43] showed that synonymous sites within the 5’-most 37 nucleotides of a coding region had the largest effect on levels of an exogenous protein being expressed off a plasmid construct (see also [39]). Our five mutations are located >100 nucleotides downstream of the start codon, so they might be expected to have a much smaller effect. A number of differences in experimental design, however, make it difficult to compare our results directly with those reported by Kudla et al. [43]. First, Kudla et al. [43] altered the codon usage of an exogenous gene located on a plasmid, while we worked with a native gene in its natural position within the genome. Second, the exogenous GFP gene used by Kudla et al. [43] does not contribute to the fitness of *E*. *coli*, so they measured gene expression rather than fitness. Third, the constructs used by Kudla et al. [43] had randomized codon usage and were, thus, quite far from the equilibrium codon usage pattern for a highly expressed gene in *E*. *coli*, while we changed only either a single nucleotide at a time or five nucleotides together, leaving the wild-type codon usage otherwise intact.

More direct comparisons can be made between our results and those reported by Lind et al. [19]. As with our system, Lind et al. [19] introduced mutations to a native gene encoding a ribosomal protein and measured fitness through competition experiments between strains carrying and not carrying the mutations. They engineered 38 strains in a close relative of *E*. *coli*, *Salmonella typhimurium*, each with a different, single synonymous mutation (plus another 88 strains with unique non-synonymous mutations). Their strains each carried only a single mutation, so the mutation-bearing strains were otherwise at wild-type codon usage. Nevertheless, there are a number of important procedural differences between our experiments and those performed by Lind et al. [19]. In our mutant strains, the entire native genome remained intact, except for one marker mutation that is selectively neutral under the experimental conditions and the single silent mutation that we wished to test. We did not include any antibiotic resistance marker as part of our final constructs. In contrast, Lind et al. [19] replaced the native gene with a construct that included both the target gene with the intended mutation plus an additional cassette carrying a gene encoding kanamycin resistance. The kanamycin resistance cassette remained in the bacterial genome in both the test and control strains. Our systems also differ in the means used to distinguish between the test strain and the wild type strain in competition experiments. We followed the general procedure pioneered by Lenski [37], in which the two strains differ in the ability to catabolize arabinose—a trait that is neutral under our competition conditions. The strains used by Lind et al. [19] in their competitions were distinguished by the presence of one of two additional exogenous genes, encoding either yellow (YFP) or cyan (CFP) fluorescent protein. They then used the different emission characteristics of those exogenous proteins to separate and count the two populations of cells using fluorescence-activated cell sorting. YFP and CFP themselves carry a fitness cost to the cells, which differs between the two versions of the fluorescent marker, requiring Lind et al. [19] to apply a mathematical correction to observed fitness values. The overall difference in the strain-marking and fitness-detection systems between our experiments and those of Lind et al. [19] also resulted in a large difference in the time course of the experiments. By virtue of being able to count a very large number of cells, the competition experiments of Lind et al. [19] ran for far fewer cell generations compared to ours. Finally, our procedures for cell growth during the competitions differed from those of Lind et al. [19]. We passaged cells at approximately 4-hour intervals in order to maintain exponential growth, while Lind et al. [19] passaged cells at 24-hour intervals and, thus, did not maintain cells in any one consistent growth phase. However, our procedure also included breaks during which growth was stopped by storage of the cultures at 2°.

Despite the substantial differences in experimental methodology, our results are quite similar to, and consistent with, those reported by Lind et al. [19]. For the three synonymous substitutions for which we found significant negative selection, our estimated selection coefficients are similar in magnitude to those reported for several synonymous mutations by Lind et al. [19]. We believe that the similarities between our results and those of Lind et al. [19] render both sets of results more compelling, precisely because they were obtained using substantially different protocols and procedures. The results from both experiments are inconsistent with the hypothesis that the strength of selection acting against a particular synonymous nucleotide is a function only of the relative abundance of the cognate tRNA. The evidence reported by Lind et al. [19] against the tRNA-abundance hypothesis is that they found no relationship between selection coefficients and the rarity of a codon as measured by the Codon Adaptation Index. They further found that the selection coefficients for silent substitutions were of generally similar magnitude to the selection coefficients observed for replacement substitutions. Our evidence against the tRNA-abundance hypothesis follows from the fact that we include two cases in which we use the same mutant codon as a replacement for the same wild-type codon, with the otherwise identical codons being separated by only a few amino acids. The tRNA-abundance hypothesis would predict that the two members of each pair should have similar selective effect, yet, for both pairs, we find substantial, significant differences in the measured selection coefficients.

It is not clear what is mediating the strong purifying selection that affects some silent sites. In a follow-up to the study of Lind et al. [19] using the same experimental system, Lind and Andersson [42] studied the role of mRNA secondary structure on determining the fitness of silent mutations. After calculating a predicted secondary structure for the mRNA, they constructed pairs of mutants that first disrupted (with a single mutation) and then restored a predicted stem pairing (with a new, complementary mutation at the paired site). They did this for nine predicted pairs of bases. Among those nine, they observed two cases for which the single mutants were selected against but the double mutant rescued wild-type fitness. We did not set up our experiment to test that particular hypothesis and, thus, we do not have any cases of disruption plus restoration of a predicted stem structure. Nevertheless, our results do not appear to be consistent with that paradigm. First, it is not clear exactly how to predict the secondary structure of the mRNA. We used the same secondary structure prediction program as did Lind and Andersson [42], but two versions of the algorithm return very different predictions for the effects of several of our introduced mutations. In addition, one of our three mutations with strong selective disadvantage was predicted to be in a single-stranded loop region. Folding effects could, of course, be responsible for the fitness effects in this study, but both more experiments designed to test that hypothesis and a better understand of the (presumably) dynamic mRNA secondary structures during translation will be required.

Firnberg et al. [44] performed a comprehensive mutagenesis of the *TEM-1* beta-lactamase gene, which confers resistance to ampicillin and is carried on a plasmid that is native to *E*. *coli*. Following growth of mixed cultures in various concentrations of ampicillin, they estimated fitness of virtually all possible silent (and replacement) substitutions by deep sequencing. The estimated error around their fitness estimates are sufficiently large to preclude measuring fitness effects on the order of the mutations in our study (*s ≈ 0*.*02*) The smallest selective effect in *TEM-1* for a silent site for which s ± the estimated error did not include 0 was s > 0.12. Nevertheless, they found significantly non-zero selective effects of silent mutations at a surprisingly high 57% of the codons in *TEM-1*, not counting the 14 codons at the 5’ end of the gene.

A different kind of study was performed by Lawrie et al. [21]. They did not modify codon usage, but instead performed genome-wide sampling of silent-site SNPs in 130 inbred strains of *Drosophila melanogaster* that originated from a single population. They found that ~20% of all silent sites in *Drosophila melanogaster* experience selection on the order of s = -0.02. This strength of selection is consistent with our estimates for strong selection against some silent mutations in *E*. *coli* and far in excess of that expected under a model of mutation-selection-drift based on interactions between the codon and the abundance of tRNAs.

### Epistasis

The mutant allele with five less-preferred silent sites exhibited fitness that was significantly lower than what would be projected from the individual-mutant fitnesses, under either an additive or multiplicative fitness model. It is not clear, however, if all five mutations contribute to the low fitness of the *rplQ*m5 allele, or only the three mutations with low individual fitness. Without understanding the actual mechanism underlying the individual fitness effects, it is difficult to suggest any specific cause for the epistatic interaction.

## Conclusions

This work demonstrates an effective method of placing single-nucleotide mutations within the wild-type *E*. *coli* genome that should be applicable to any chloramphenicol-sensitive, ampicillin-sensitive bacterium supporting the Ts replication of a plasmid vector (e.g., pIB307) and a competition protocol for measuring selective effects to approximately 10^−3^. We have demonstrated that several synonymous mutations confer strong selective disadvantage under our culture conditions (three of the five synonymous positions we tested), and that negative, synergistic epistasis occurs when all five synonymous mutations are introduced together as a single allele.

## Supporting Information

S1 File Supporting Information. **Table A.** Primers and synthesized oligomers used in this study. **Table B.** Plasmids used and constructed in this work. **Table C.** Strains of *E*. *coli* used or constructed in this work with relevant genotypes or phenotypes and derivations. **Table D.** Data for competition experiment (Competition 20070418) for the wild-type K-12 strain MG1655 vs. Ara^-^ HJD2 strain. Data includes colony counts, initial and final population sizes, doublings and relative fitness of replicates from the competition experiment (n = 5) and statistical information. **Table E.** Data for competition experiment (Competition 20070503) for the wild-type K-12 strain MG1655 vs. Ara^-^ HJD2 strain. **Table F.** Data for competition experiment (Competition 20071024) for the wild-type K-12 strain MG1655 vs. Ara^-^ HJD2 strain. Because of the greater variation in number of doublings among the replicates, this one competition experiment was run for 15 growth periods to assure at least 100 generations for all replicates. **Table G.** Data for competition experiment (Competition 2010126) for the wild-type K-12 strain MG1655 vs. Ara^-^ HJD2. **Table H.** Data for competition experiment (Competition 20080414) for the wild-type K-12 strain MG1655 vs. HDJ4e with *rplQ*m5. **Table I.** Data for competition experiment (Competition 20080313) for the wild-type K-12 strain MG1655 vs. HDJ4h with *rplQ*m5. **Table J.** Data for competition experiment (Competition 20080325) for the wild-type K-12 strain MG1655 vs. HDJ4i with *rplQ*m5. **Table K.** Data for competition experiment (Competition 20080821) for the wild-type K-12 strain MG1655 vs. Ara^-^/*rplQ* revertant wild type HJD6. **Table L.** Data for competition experiment (Competition 20091111) for the wild-type K-12 strain MG1655 vs. Ara^-^/*rplQ*mL44 HJD13. **Table M.** Data for competition experiment (Competition 20091130) for the wild-type K-12 strain MG1655 vs. Ara^-^/*rplQ*mL38 HJD14. **Table N.** Data for competition experiment (Competition 20100113) for the wild-type K-12 strain MG1655 vs. Ara^-^/*rplQ*mK40 HJD15. **Table O.** Data for competition experiment (Competition 20091207) for and the wild-type K-12 strain MG1655 vs. Ara^-^/*rplQ*mK42 HJD16. **Table P.** Data for competition experiment (Competition 20100104) for and the wild-type K-12 strain MG1655 vs. Ara^-^/*rplQ*mV47 HJD17. **Info A.** Calculating the variance of the calculated relative fitness from 5 LPSCs through propagation of error. **Info B.** Calculating the minimum detectable selection coefficient.
(DOCX)
