# Supplementary material for: Mutations to Less-Preferred Synonymous Codons in a Highly Expressed Gene of Escherichia coli: Fitness and Epistatic Interactions
Source: PLoS One. 2016 Jan 4;11(1):e0146375. doi: 10.1371/journal.pone.0146375 (PMC4699635; doi:10.1371/journal.pone.0146375)
Supplement: S1 File — Table A. Primers and synthesized oligomers used in this study. Table B. Plasmids used and constructed in this work. Table C. Strains of E. coli used or constructed in this work with relevant genotypes or phenotypes and derivations. Table D. Data for competition experiment (Competition 20070418) for the wild-type K-12 strain MG1655 vs. Ara- HJD2 strain. Data includes colony counts, initial and final population sizes, doublings and relative fitness of replicates from the competition experiment (n = 5) and statistical information. Table E. Data for competition experiment (Competition 20070503) for the wild-type K-12 strain MG1655 vs. Ara- HJD2 strain. Table F. Data for competition experiment (Competition 20071024) for the wild-type K-12 strain MG1655 vs. Ara- HJD2 strain. Because of the greater variation in number of doublings among the replicates, this one competition experiment was run for 15 growth periods to assure at least 100 generations for all replicates. Table G. Data for competition experiment (Competition 2010126) for the wild-type K-12 strain MG1655 vs. Ara- HJD2. Table H. Data for competition experiment (Competition 20080414) for the wild-type K-12 strain MG1655 vs. HDJ4e with rplQm5. Table I. Data for competition experiment (Competition 20080313) for the wild-type K-12 strain MG1655 vs. HDJ4h with rplQm5. Table J. Data for competition experiment (Competition 20080325) for the wild-type K-12 strain MG1655 vs. HDJ4i with rplQm5. Table K. Data for competition experiment (Competition 20080821) for the wild-type K-12 strain MG1655 vs. Ara-/rplQ revertant wild type HJD6. Table L. Data for competition experiment (Competition 20091111) for the wild-type K-12 strain MG1655 vs. Ara-/rplQmL44 HJD13. Table M. Data for competition experiment (Competition 20091130) for the wild-type K-12 strain MG1655 vs. Ara-/rplQmL38 HJD14. Table N. Data for competition experiment (Competition 20100113) for the wild-type K-12 strain MG1655 vs. Ara-/rplQmK40 HJD15. Table O. Data for c [file pone.0146375.s001.docx]

**Supporting Information**

**Table A.** Primers and synthesized oligomers used in this study.

**Table B.** Plasmids used and constructed in this work.

**Table C.** Strains of *E. coli* used or constructed in this work with relevant genotypes or phenotypes and derivations.

**Table D.** Data for competition experiment (Competition 20070418) for the wild-type K-12 strain MG1655 vs. Ara^-^ HJD2 strain. Data includes colony counts, initial and final population sizes, doublings and relative fitness of replicates from the competition experiment (n = 5) and statistical information.

**Table E.** Data for competition experiment (Competition 20070503) for the wild-type K-12 strain MG1655 vs. Ara^-^ HJD2 strain.

**Table F.** Data for competition experiment (Competition 20071024) for the wild-type K-12 strain MG1655 vs. Ara^-^ HJD2 strain. Because of the greater variation in number of doublings among the replicates, this one competition experiment was run for 15 growth periods to assure at least 100 generations for all replicates.

**Table G.** Data for competition experiment (Competition 2010126) for the wild-type K-12 strain MG1655 vs. Ara^-^ HJD2.

**Table H. Data for competition experiment (Competition 20080414) for the wild-type K-12 strain MG1655 vs. HDJ4e with *rplQ*m5.**

**Table I. Data for competition experiment (Competition 20080313) for the wild-type K-12 strain MG1655 vs. HDJ4h with *rplQ*m5.**

**Table J. Data for competition experiment (Competition 20080325) for the wild-type K-12 strain MG1655 vs. HDJ4i with *rplQ*m5.**

**Table K. Data for competition experiment (Competition 20080821) for the wild-type K-12 strain MG1655 vs. Ara^-^/*rplQ* revertant wild type HJD6.**

**Table L. Data for competition experiment (Competition 20091111) for the wild-type K-12 strain MG1655 vs. Ara^-^/*rplQ*mL44 HJD13.**

**Table M. Data for competition experiment (Competition 20091130) for the wild-type K-12 strain MG1655 vs. Ara^-^/*rplQ*mL38 HJD14.**

**Table N. Data for competition experiment (Competition 20100113) for the wild-type K-12 strain MG1655 vs. Ara^-^/*rplQ*mK40 HJD15.**

**Table O. Data for competition experiment (Competition 20091207) for and the wild-type K-12 strain MG1655 vs. Ara^-^/*rplQ*mK42 HJD16.**

**Table P. Data for competition experiment (Competition 20100104) for and the wild-type K-12 strain MG1655 vs. Ara^-^/*rplQ*mV47 HJD17.**

**Info A. Calculating the variance of the calculated relative fitness from 5 LPSCs through propagation of error**

Originally, it was anticipated that the individual selection coefficients were likely very small with respect to that achieved with the 5 LPSCs within *rplQ*, or any gene; however, with selection coefficients of -0.0234, -0.0267, and -0.0262, a fairly large resultant selection coefficient was building up whether the multiplicative or the additive method of combining the individual selection coefficients was used. It was, therefore, considered necessary to apply an approximation for the propagation of error for each calculation to estimate the variance of the resultant expected fitness. The variance approximation, V_w_, for the accumulated fitness effects was calculated as

$V_{w}=\sum\left(\frac{\partial w}{\partial n}\right)^{2}V_{n}$ (Equation A.1)

where V_n_ is the variance for each individual fitness value. This approximation is considered valid for any function *w = f(x,y,z,…)* for which the individual variances are not great (Peters, Hayes, & Hieftje 1974, pp. 19-22; Bevington & Robinson 2003, p. 41). Hence for a function *w* = f(x,y,z,u,t), $V_{w}=\left(\frac{\partial w}{\partial x}\right)^{2}V_{x}+\left(\frac{\partial w}{\partial y}\right)^{2}V_{y}+\left(\frac{\partial w}{\partial z}\right)^{2}V_{z}+\left(\frac{\partial w}{\partial u}\right)^{2}V_{u}+\left(\frac{\partial w}{\partial t}\right)^{2}V_{t}$.(Equation S1.2)

Here *V_w_* is the calculated variance for the calculated fitness and *V_x_, V_y_, V_z_, V_u_, and V_t_* are the variances for each of the 5 singly mutated substrains. For the calculation of variance for the additive formula $w=1+\sum_{i=1}^{n} s_{i}$, $\frac{\partial w}{dx}=\frac{\partial w}{dy}=\frac{\partial w}{\partial z}=\frac{\partial w}{\partial u}=\frac{\partial w}{\partial t}=1$, so

*V_w_ =V_mL44_ + V_mL38_ + V_mK40_ + V_mK42_ + V_mV47_.* (Equation A.3)

For the calculation of variance for multiplicative formula$w=\prod_{i=1}^{n} \left(1+s_{i}\right)^{n}$, $\frac{\partial w}{\partial x}=yzut=\frac{w}{x}$. By this, the estimated variance of the combined fitness according to the multiplicative formula is

$V_{w}=\left(\frac{w}{x}\right)^{2}V_{x}+\left(\frac{w}{y}\right)^{2}V_{y}+\left(\frac{w}{z}\right)^{2}V_{z}+\left(\frac{w}{u}\right)^{2}V_{u}+\left(\frac{w}{t}\right)^{2}V_{t}$ (Equation A.4)

where *w* is the calculated estimated fitness, and x, y, z, u, and t are the relative fitnesses of the individual mutations. Because there are 5 values, 3 of which being negative and 2 positive and the issue of spontaneous acclimating mutations discussed above, values for the multiplicative and additive models were figured two ways: (1) calculation with all 5 selection coefficients with the variance calculated with all 5 individual variances, and (2) calculation with only the 3 negative selection coefficients, but still calculating the variance with all 5 individual variances. The calculated additive fitness of the five LPSCs was 0.9336 while that of the three exhibiting negative selection coefficients was 0.9237. The estimated standard deviation is ±0.0075. The calculated multiplicative fitness of the 5 LPSCs is 0.9348 while that of the 3 with negative selection coefficients was 0.9256. The estimated standard deviation for the multiplicative fitness is ±0.0071. These data along with the ±1 standard deviation range and the 95% confidence intervals for the *rplQ*m5 substrains and the singly mutated substrains are shown in Fig 3; however, for the calculated fitness values, the ±2 standard deviation ranges are used rather than the confidence intervals. The 95% confidence interval for the *rplQ*m5 substrains HDJ4e, h, and i were all below the 2 standard deviation range of any of the methods used to calculate estimated additive or multiplicative fitness.

**Info B. Calculating the minimum detectable selection coefficient**

The minimum detectable difference equation for the two-sample t-test (Zar, 1999) for α = 0.05 and β = 0.10 (for Power = 0.90) is:

δ  > (2s_p_^2^/n)^0.5^ (t_α(2),ν_ + t_β(1),ν_). (Equation B.1)

In this equation, the pooled variance, s_p_^2^ = (SS_1_ + SS_2_)/ (ν_1_ + ν_2_)

in which SS_1_ = sum of squares for the replicate doublings, n = the number of replicates, and ν_1_ = the degrees of freedom for the wild type, and SS_2_ & ν_2_ are those, respectively, for the mutant. The t values are from the respective values from critical values of the t distribution for which ν = ν_1_+ν_2_.

The minimum detectable difference δ is in units of doublings;

D_m_ = D_wt_ + δ or δ = D_m_ – D_wt_ (Equation B.2),

in which D is the number of doublings and the subscripts m and wt refer to mutant and wild type. Actually, since δ is based on the sum of squares, δ could also equal D_wt_ – D_m_ .

Since D = log_2_(N_f_/N_0_), (Equation B.3)

in which N_f_ = the final population size and N_0_ = the initial population sizes, combining equations (S2.2) and (S2.3) results in:

log_2_(N_fm_/N_0m_) - log_2_(N_fwt_/N_0wt_) = δ. (Equation B.4)

Therefore,

log_2_N_fm_ - log_2_N_0m_ - (log_2_N_fwt_ – log_2_N_0wt_) = δ. (Equation B.5)

Assuming that N_0_ of the mutant = N_0_ of the wild type for ease of calculations,

log_2_N_0m_ = log_2_N_0wt_. Then,

log_2_N_fm_ - log_2_N_fwt_ = δ, (Equation B.6)

so

log_2_(N_fm_ / N_fwt_) = δ. (Equation B.7)

By definition of logarithms,

2^δ^ = N_fm_ / N_fwt_. (Equation B.8)

At this point, another concept is needed—population differential, PD. PD will be defined as the ratio of the final population of the mutant to that of the wild type at the end of the competition period, or more precisely,

*PD* = (N_fm_/N_0m_) / (N_fwt_/N_0wt_). (Equation B.9)

Again, assuming the same size of initial populations (N_0m_ = N_0wt_), this equation simplifies to

*PD* = N_fm_/N_fwt_. (Equation B.10)

Because

N_fwt_ = N_0_•2^n^ , where n is the number of doublings or generations, (Equation B.11)

and the effect of selection over generations (or doublings) is (1+s)^n^ of the wild-type population,

N_fm_ = N_0_(1+s)^n^•2^n^, (Equation B.12)

the following statement can be made:

*PD* = (N_0_(1+*s*)^n^•2^n^) / (N_0_•2^n^) = (1+s)^n^. (Equation B.13)

Combining the equalities in Equations S2.8, S2.10, and S2.13, one arrives at

*PD* = 2^δ^ = (1+*s*)^n^ (Equation B.14)

which can be used to estimate the minimum difference in population size and, therefore, colony counts, required to detect selection operating.

What one really would like to know, though, is what is the selection coefficient *s* necessary to give an over or under performance of 2^δ^ in population size over n generations. Taking the logarithm of both sides of Equation S2.14 for *PD*, (1+*s*)^n^ = 2^δ^ one gets

n•log_2_(1+s) = δ•log_2_ 2. (Equation B.15)

Since log_2_ 2 = 1, one can divide by n, the number of generations to get:

log_2_(1+s) = (δ•log_2_2)/n = δ/n. (Equation B.16)

Since log_2_2 = 1, log_2_(1 + *s*) = δ/n, resulting in

2^δ/n^ = 1 + *s*. (Equation B.17)

Solving for *s*,

*s* = 2^δ/n^ – 1. (Equation B.18)

One will end up with *s* = 2^δ/n^ – 1 whether one starts with D_wt_ = D_m_ + δ or D_m_ = Dwt + δ.

2^δ/n^ will always be greater than 1, unless the minimum detectable difference is 0, resulting in 2^δ/n^ = 2^0^ = 1, and s = 1-1 = 0, obviously an unreachable limit. Basically, δ/n cannot be a negative number because all the numbers used in calculating δ (based on the sum of squares of the differences between the number of doublings for both the wild type and mutant strains) and n (the number of generations) are positive. As a result, one gets the magnitude of the difference, not directional difference (+ or -). Since *s* is considered positive when advantageous and negative when deleterious—*i.e.,* *PD* = (1 + *s*)^n^— in this work, directional selection can be denoted by the reciprocal value of *PD*, 2^-δ`^, for a negative value for the selection coefficient. With this adjustment to show directionality with deleterious mutations,

s = 2^-δ/n^ -1. (Equation B.19)

**Summarizing:**

Minimum detectable selective coefficient: *s* = 2^δ/n^ -1 (positive s) OR *s* = 2^-δ/n^ -1 (negative s).

Population Differential: *PD* = (1+*s*)^n^ = 2^δ^ (for positive selection) OR = 2^-δ^ (for negative s).
